# The APOE ε2 allele is associated with risk of neovascular age-related macular degeneration in a Russian cohort

**DOI:** 10.18699/vjgb-26-83

**Published:** 2026-09

**Authors:** .S. O Kozhevnikova, I.F. Nikulich, A.S. Derbeneva, V.A. Devyatkin, N.G. Kolosova, A.Zh. Fursova

**Affiliations:** Institute of Cytology and Genetics of the Siberian Branch of the Russian Academy of Sciences, Novosibirsk, Russia; State Novosibirsk Regional Clinical Hospital, Novosibirsk, Russia Novosibirsk State Medical University, Novosibirsk, Russia; State Novosibirsk Regional Clinical Hospital, Novosibirsk, Russia Novosibirsk State Medical University, Novosibirsk, Russia; Institute of Cytology and Genetics of the Siberian Branch of the Russian Academy of Sciences, Novosibirsk, Russia; Institute of Cytology and Genetics of the Siberian Branch of the Russian Academy of Sciences, Novosibirsk, Russia; Institute of Cytology and Genetics of the Siberian Branch of the Russian Academy of Sciences, Novosibirsk, Russia State Novosibirsk Regional Clinical Hospital, Novosibirsk, Russia Novosibirsk State Medical University, Novosibirsk, Russia

**Keywords:** age-related macular degeneration, APOE, rs429358, rs7412, response to anti-VEGF, возрастная макулярная дегенерация, APOE, rs429358, rs7412, ответ на анти-VEGF-терапию

## Abstract

The APOE ε2 allele is associated with risk of neovascular age-related macular degeneration in a Russian cohort

## Abstract (ru)

Аллель APOE ε2 ассоциирован в российской когорте с риском неоваскулярной возрастной макулярной дегенерации

## Introduction

Age-related macular degeneration (AMD) is a progressive multifactorial blinding disease, representing the leading cause of visual impairment in an aging population. AMD is characterized by atrophy of the retinal pigment epithelium (RPE), accumulation of drusen, inflammation, and photoreceptor loss in the macular region of the eye. Genetic susceptibility, along with age and lifestyle, constitute the primary risk factor of AMD, and can influence the disease phenotype and treatment outcome. Among multiple genes and/or single nucleotide polymorphisms (SNPs) associated with AMD, genome-wide association studies have identified lipid metabolism pathway genes, contributions of rare and common variants of which are highlighted in a large genome-wide association study (Fritsche et al., 2016; Fernández-Vega et al., 2020).

Accordingly, numerous data confirm the involvement of lipid metabolism violations in the pathogenesis of AMD including: (1) drusen, the hallmark histopathologic feature of AMD, are composed of lipids; (2) polymorphisms of genes involved in lipid homeostasis are associated with increased odds of AMD; (3) metabolomics studies show that patients with AMD have alterations in metabolites from lipid pathways, and (4) alterations in serum lipid profiles as a reflection of systemic dyslipidemia are associated with AMD (Lin et al., 2022). Apolipoprotein E (ApoE) is a transport protein of lipid and cholesterol, playing a pivotal role in lipid metabolism in the peripheral circulation, in the brain and in the re-innervation process following nervous system injury. ApoE has the ability to bind to membrane receptors, mediating lipid transfer between circulating lipoproteins and tissues. Additionally, ApoE nonspecifically binds to lipophilic inflammatory components, contributing to their clearance and participating in the innate immune response (Huebbe, Rimbach, 2017).

Three major isoforms are known to exist in the ApoE protein. ApoE2, ApoE3, and ApoE4 are the products of three alleles (ε2, ε3, and ε4) at a single gene locus defined by two SNPs (rs429358 and rs7412): ApoE3 has cysteine in position 112, and arginine, in position 158, ApoE2 has cysteine in both positions, and ApoE4 has arginine in both positions (Arg112, Cys158 configuration is extremely rare). There are six genotypes in the general population: three homozygous (APOE ε2/ε2, APOE ε3/ε3, and APOE ε4/ε4) and three heterozygous (APOE ε2/ε3, APOE ε2/ε4, and APOE ε3/ε4). The ε3 allele is the most common and is found in 77 % of the general population. Meanwhile, the ε2 allele is found in 8 % of the general population, and the ε4 allele is found in 15 % of the general population. However, the frequency of APOE alleles varies between different ethnic groups (Chan et al., 2023).

The APOE gene is a major genetic risk factor for development of both AMD and late-onset Alzheimer’s disease (AD) – the leading cause of dementia in the elderly. Despite the different clinical manifestations of these diseases, growing evidence suggests that they share common pathways in their pathogenesis including inflammation, oxidative stress, and impaired autophagy. It is also worth noting that ApoE is a ubiquitous component of drusen, a hallmark of early AMD, and abundantly present in amyloid plaques in the AD brain (Wisniewski, Drummond, 2020). There are many data indicating that ApoE variants that have a protective association against AD are also associated with a simultaneous high risk of AMD. Therefore, it is known that the ε4 allele reduces the risk of AMD and at the same time is the major risk factor for AD. On the contrary, individuals with the ε2 allele have a higher risk of AMD but a reduced risk of AD (Liuska et al., 2023).

There are, however, conflicting results. Recently, Fernández- Vega et al. (2020) described a protective role the APOE ε2 allele has against wet AMD in the Northern Spanish population. A meta-analysis integrating several recent large-sample studies to verify the effect of APOE polymorphisms on AMD subtypes revealed a significantly protective role of ε4 on each subtype of AMD, but no supportive evidence of the association of ε2 with AMD (Xiying et al., 2017). On the contrary, according to the data of Gupta and Chawla (2025), the APOE ε4 allele may be a risk factor for AMD in the Indian population. At the same time in a large, longitudinal cohort of more than 100 000 Europeans, Rasmussen et al. (2023) revealed that the ε2 allele has an effect on AMD and confirmed an increased risk of AMD in people with this genotype. No evidence was received to support an association of APOE gene polymorphisms with early or exudative AMD in the Chinese (Sun et al., 2011) and Brazilian populations (Viturino et al., 2021). According to data of Adams et al. (2012), in the Australian population, associations of ε2ε2 and ε2ε4 with early AMD varied by smoking status.

These results demonstrate the importance of potential modifiers of association between APOE polymorphism and AMD and highlight its significant effects on this association that should be taken into account in further studies. Thus, the association of the ε2 allele with AMD is controversial, and their roles in different subtypes of the disease need to be properly explored in different populations for a better understanding of AMD pathophysiology. Our goal was to study the effect of APOE alleles on the risk of developing AMD and response to anti-VEGF treatment in patients of Russian ethnicity from the Western Siberian population.

## Materials and methods

Study population and ethical approval. This study was conducted at the Ophthalmology Department of the Novosibirsk Regional Clinical Hospital (clinical assessment) and the Institute of Cytology and Genetics, SB RAS (genetic analysis). The study was performed in accordance with the tenets of the Declaration of Helsinki and was approved by the local Ethics Committee. All participants provided written informed consent.

The study included 315 patients diagnosed with neovascular age-related macular degeneration (nAMD) and a control group of 317 individuals without AMD. Inclusion criteria for the nAMD group were: age over 50 years, a diagnosis of nAMD with active macular neovascularization confirmed by the presence of subretinal and/or intraretinal fluid on optical coherence tomography (OCT), and provision of informed consent. A subgroup of 275 treatment-naïve patients was selected for the anti-VEGF therapy response analysis. Exclusion criteria were: a history of laser coagulation, previous intravitreal pharmacotherapy, geographic atrophy, signs of intraocular inflammation, or any prior vitreoretinal surgery.

The control group consisted of individuals over 50 years of age with no clinical signs of AMD (e. g., soft or hard drusen, geographic atrophy, RPE detachment, macular fibrosis, or pigmentary abnormalities). Patients with senile cataract were included in the control group.

Clinical examination and anti-VEGF treatment protocol. Peripheral blood samples were collected from all participants in the morning after fasting into EDTA-containing vacuum tubes. Patients in the therapy subgroup (n = 275) received three loading doses of aflibercept (0.5 mg/0.05 mL) administered intravitreally at 4-week intervals. Injections were performed under topical epibulbar anesthesia (Alcaine) using a 27-gauge needle inserted at least 3 mm from the limbus. Treatment efficacy was assessed through clinical and instrumental examination before the initiation of therapy and after the third loading dose. This included: best-corrected visual acuity (BCVA) measured using an ETDRS (Early Treatment Diabetic Retinopathy Study) chart, central retinal thickness (CRT) measured by optical coherence tomography (OCT). The treatment response was classified by clinicians at the Novosibirsk Regional Clinical Hospital as optimal (good), partial (weak), or no response, based on the dynamics of BCVA and CRT changes, approximating the criteria proposed by Amoaku et al. (2015).

DNA extraction and APOE genotyping. Genomic DNA was isolated from whole blood using a diaGene (Russia) DNA extraction kit according to the manufacturer’s protocol. DNA samples were diluted to a concentration of 8 ng/μL and stored at –20 °C. APOE genotyping for the rs429358 and rs7412 polymorphisms was performed using the allele-specific PCR with the TaqMan probes method described by Zhong et al. (2016). The genotyping assay comprised three separate reactions to identify the ε2, ε3, and ε4 alleles. Each 25 μL PCR reaction mixture contained: 12.5 μL of 2× BioMaster HS-qPCR mix (Biolabmix, Russia), containing HS-Taq DNA polymerase, dNTPs, PCR buffer, and Mg2+, 0.5 μg of each forward and reverse APOE primer, APOE TaqMan probe, genomic DNA template, water to a final volume of 25 μL. The amplification protocol was as follows: initial polymerase activation at 95 °C for 5 min, 40 cycles of denaturation at 95 °C for 15 s, and annealing/ extension at 64 °C for 1 min.

Statistical analysis was performed using StatTech v. 4.0.6 (StatTech LLC, Russia). The normality of quantitative data distribution was assessed using the Kolmogorov–Smirnov test. Data are presented as mean (M) ± standard deviation (SD) or median (Me) with interquartile range (Q1–Q3) and 95% confidence intervals (95% CI), as appropriate. Categorical data are described using absolute values and percentages. Comparisons of percentages in contingency tables were performed using Pearson’s chi-square test. A p-value of < 0.05 was considered statistically significant.

## Results

**APOE genotype and allele distribution**

Genotyping of the APOE gene polymorphisms (rs429358 and rs7412) was performed in 315 patients with neovascular agerelated macular degeneration (nAMD) and 317 control subjects using the allele-specific PCR method described by Zhong et al. (2016). The genotype distributions for both polymorphisms were in Hardy–Weinberg equilibrium in all groups.

The frequencies of the resulting ApoE isoforms (ε2, ε3, ε4) are summarized in Table 1. The overall allelic and genotypic frequencies of the rs429358 and rs7412 variants did not differ significantly between the nAMD and control groups in a logistic regression analysis adjusted for age and sex.

**Table 1.**
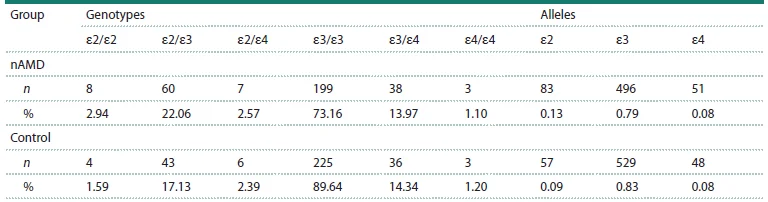
The frequency of genotypes and alleles of the APOE gene in the nAMD and control groups

However, a subsequent analysis focusing on the ApoE isoforms revealed a significant association. The frequency of the ε2 allele was significantly higher in nAMD patients compared to the control group (0.13 vs. 0.09, p = 0.018, likelihood ratio chi-square test). Carriers of the ε2 allele had a 1.5-fold increased risk of nAMD compared to carriers of other allelic variants (OR = 1.536; 95% CI: 1.075–2.195) (Table 2). These results indicate a moderate association of the APOE ε2 allele with nAMD development in the Russian population.

**Table 2.**
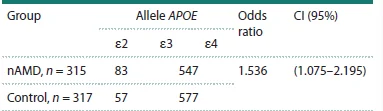
Analysis of the association of carriage of the ε2 allele with the risk of AMD

**Association between APOE genotype and treatment response**

The association between APOE genotype and variability in treatment response was assessed in a cohort of 275 nAMD patients (275 eyes). Patient clinical characteristics are presented in Table 3. The majority of patients presented with type 1 (occult) macular neovascularization (52.4 %, n = 144), followed by type 2 (44.0 %, n = 121) and type 3 (3.6 %, n = 10).

**Table 3.**
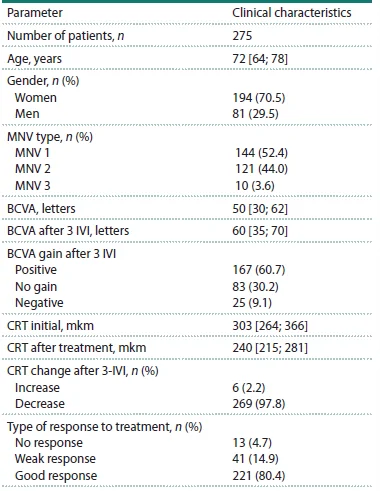
Clinical characteristics of patients

Following three loading doses of anti-VEGF therapy, patients were categorized based on treatment response: 221 (80.4 %) had an optimal response, 41 (14.9 %) had a partial response, and 13 (4.7 %) were non-responders. No statistically significant associations were found between the type of response and patient sex or age ( p > 0.05)

At diagnosis, the median central retinal thickness (CRT) was 294 μm (IQR: 238–399 μm). After three loading doses, 269 patients (97.8 %) showed a reduction in CRT, with a median decrease of 22 % to 236 μm (IQR: 213–267 μm). A positive dynamic in best-corrected visual acuity (BCVA) was observed in 167 eyes (60.7 %). The median BCVA improved from 32 letters (IQR: 30–50) at baseline to 60 letters (IQR: 35–75) in the optimal response group after loading doses. A significant increase in BCVA and a reduction in CRT were observed in the early treatment phase ( p < 0.05). However, at baseline, there were no statistically significant differences in BCVA ( p = 0.306) or CRT ( p = 0.928) between patients who later demonstrated an optimal response and those with a partial or absent response.

We hypothesized that APOE genotype might influence interindividual variability in response to therapy. However, Pearson’s chi-square analysis showed no significant association between the studied APOE alleles or genotypes and the type of response to anti-VEGF therapy (Table 4).

**Table 4.**
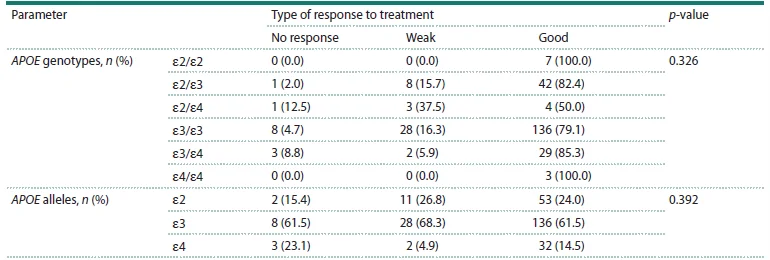
Distribution of APOE genotypes and alleles in groups with different therapeutic outcomes of anti-VEGF therapy

## Discussion

This is the first study to investigate the association of APOE ε2, ε3, and ε4 alleles with both the risk of AMD and the response to anti-VEGF therapy in a Russian population from Western Siberia. Our findings confirm a significant association between the APOE ε2 allele and an increased risk of developing neovascular AMD. However, we found no statistically significant link between APOE polymorphisms and the short-term anatomical and functional response to anti-VEGF treatment after the loading phase. Previously, the ApoE ε2/ε3/ε4 isoforms have been reported to be associated with the risk of developing AMD: in several studies, the ε4 allele was associated with a decreased risk, and the ε2 allele, with an increased risk (Adams et al., 2012).

Apolipoprotein E is a key protein in lipoprotein metabolism. It exists in three major isoforms, defined by amino acid substitutions at positions Cys112Arg and Arg158Cys, which are determined by two single-nucleotide polymorphisms (rs429358 and rs7412). These ApoE isoforms are encoded by three alleles: ε2 (rs429358*T/rs7412*T), ε3 (rs429358*T/rs7412*C), and ε4 (rs429358*C/rs7412*C). ApoE is expressed in the retina and has been identified within drusen in AMD patients (Abyadeh et al., 2023). In numerous population-based studies, the APOE ε4 variant has been associated with a protective effect against AMD, whereas the ε2 allele has been linked to an increased risk of the disease (Hu et al., 2021).

The pivotal role of anti-VEGF agents in managing neovascular AMD is undeniable, having revolutionized the prevention of severe vision loss. Nonetheless, a subset of patients, approximately 10–30 % (Fursova et al., 2023), exhibit a suboptimal response and continue to experience vision deterioration. The underlying causes for this treatment resistance remain unclear, with genetic predisposition considered a potential determinant (Kozhevnikova et al., 2023).

Given the high prevalence of AMD and the substantial socio-economic burden associated with long-term anti- VEGF therapy, the ability to predict treatment outcomes is of paramount clinical importance. We hypothesized that APOE genotype could influence the considerable individual variability in treatment response. This premise is supported by several international studies investigating the association between ApoE and AMD biomarkers (Gupta, Chawla, 2025). However, an analysis of the relationship between APOE genotype, the risk of neovascular AMD, and the response to anti-VEGF therapy had not been previously conducted within a Russian population.

We focused on the short-term response following the initial loading doses of anti-VEGF therapy, as this period is considered a critical window for assessing the maximum potential morphological and functional response. Analyzing this phase was hypothesized to best reveal the extent of genetic influence on subsequent long-term outcomes. While some studies have demonstrated that the most significant improvements occur within the first three months of treatment, our investigation in the West Siberian cohort did not identify APOE genotype as a significant predictor of outcome at this stage. This lack of association concerning the treatment response contrasts with findings from other studies. For instance, research by Wickremasinghe et al. (2011) and Bakbak et al. (2016) reported that the APOE ε4 allele was linked to better visual outcomes following anti-VEGF treatment. The disparity between our results and those in the literature could be attributed to several factors. The ethnic variation in APOE allele and genotype distribution is well-documented, suggesting that the relationship between APOE and AMD pathophysiology may be population-specific (Ramadan et al., 2019).

Furthermore, the influence of ApoE might become apparent only over a longer observation period or under different treatment regimens, such as personalized dosing protocols, as suggested by other authors. Another critical consideration is the treatment initiation timeline. A significant delay often exists between diagnosis and the start of therapy, which can drastically diminish the potential for visual improvement and might obscure underlying pharmacogenetic associations (Kozhevnikova et al., 2023).

The biological plausibility of ApoE’s role in AMD is supported by its involvement in lipid metabolism. ApoE is detected in drusen, the earliest hallmarks of AMD, and is crucial for lipid transport across cell membranes, being highly expressed by the retinal pigment epithelium (RPE) (Anderson et al., 2001). Evidence from murine models indicates that the ε2 allele is associated with age-related accumulation of subretinal phagocytes and exacerbated choroidal neovascularization, while the ε4 allele shows protective effects (Levy et al., 2015). This aligns with population studies where the ε4 allele often confers a protective effect against AMD development, and ε2 increases risk, a pattern consistent with our findings regarding disease susceptibility (Adams et al., 2012).

APOE polymorphisms can influence treatment outcomes in AMD patients. In patients with neovascular AMD, the presence of the APOE ε4 allele conferred significantly better visual outcomes after anti-VEGF treatment (Wickremasinghe et al., 2011). The results of a study by Bakbak et al. (2016) also showed improvement of visual activity in AMD patients carrying the ε4 allele and indicated an association between the ε4 allele and visual improvement in AMD patients using ranibizumab, whereas no association with treatment was observed for the ε2 allele.

## Conclusion

Our study revealed a statistically significant association between the ε2 allele of APOE and the development of neovascular AMD in the Russian population of Western Siberia. No association was found between APOE genotype and short-term response to anti-VEGF therapy (after three intravitreal injections). Considering the rising prevalence of AMD, further pharmacogenetic studies are essential to elucidate potential genetic markers that could guide personalized treatment strategies.

## Conflict of interest

The authors declare no conflict of interest.
